# Early detection of bipolar disorders and treatment recommendations for help-seeking adolescents and young adults: Findings of the Early Detection and Intervention Center Dresden

**DOI:** 10.1186/s40345-021-00227-3

**Published:** 2021-07-02

**Authors:** Julia Martini, Karolina Leopold, Steffi Pfeiffer, Christina Berndt, Anne Boehme, Veit Roessner, Paolo Fusar-Poli, Allan H. Young, Christoph U. Correll, Michael Bauer, Andrea Pfennig

**Affiliations:** 1 Department of Psychiatry and Psychotherapy, Carl Gustav Carus University Hospital, Technische Universität Dresden, Fetscherstraße 74, 01307 Dresden, Germany; 2 Department of Psychiatry, Psychotherapy and Psychosomatics, Vivantes Klinikum Am Urban, Berlin, Germany; 3 Department of Child- and Adolescent Psychiatry and Psychotherapy, Carl Gustav Carus University Hospital, Technische Universität Dresden, Dresden, Germany; 4 Early Psychosis: Intervention and Clinical-Detection (EPIC) Lab, Institute of Psychiatry, Psychology and Neuroscience, King’s College London, London, UK; 5 Department of Brain and Behavioral Sciences, University of Pavia, Pavia, Italy; 6 Department of Psychological Medicine, Institute of Psychiatry, Psychology and Neuroscience, King’s College London & South London and Maudsley NHS Foundation Trust, Bethlem Royal Hospital, London, UK; 7 Department of Child- and Adolescent Psychiatry and Psychotherapy, Charité Universitätsmedizin, Berlin, Germany; 8 Department of Psychiatry, The Zucker Hillside Hospital, Northwell Health, Glen Oaks, NY USA; 9 Donald and Barbara Zucker School of Medicine At Hofstra/Northwell, Department of Psychiatry and Molecular Medicine, Hempstead, NY USA

## Abstract

**Background:**

Early identification and intervention of individuals with risk factors for or subtle prodromal symptoms of bipolar disorders (BD) may improve the illness course and prevent adverse long-term consequences.

**Methods:**

We examined sociodemographic, clinical and psychopathological characteristics of help-seeking adolescents and young adults who consulted the Early Detection and Intervention Center Dresden at the University of Dresden (Germany) and presented with or without pre-defined at-risk criteria for BD. The standardized diagnostic procedure for all help-seeking youth included a comprehensive psychiatric history and a structured clinical interview. When BD at-risk state was suspected, early detection instruments (EPI*bipolar*, BPSS-FP) were applied. Treatment recommendations were formulated in multi-professional case conferences.

**Results:**

Out of 890 help-seeking persons between 05/2009 and 04/2018, 582 (65%) completed the diagnostic process. Of these, 24 (4%) had manifest BD and 125 (21%) fulfilled at-risk BD criteria (age = 23.9 ± 0.6 years, female = 62%). Of the pre-defined main risk factors, family history for BD was reported in 22% of the at-risk persons, (hypo-)mania risk state in 44%, and increasing cyclothymic mood swings with increased activity in 48%. The most common secondary risk factors were decreased psychosocial functioning (78%), lifetime diagnosis of depressive disorder (67%) and specific sleep/circadian rhythm disturbances (59%). Substance use was very common in subjects at-risk for BD (cannabis = 50%, alcohol = 33%) and highest in patients with BD (cannabis = 75%, alcohol = 40%). Psychiatric treatment history, including psychopharmacological therapy, was similar between the groups, while treatment recommendations differed, with more advice for psychotherapy and antidepressants in the at-risk group with a lifetime diagnosis of depression and more advice for specialized BD treatment including mood stabilizers in patients with BD.

**Conclusion:**

This analysis on the phenomenology of different BD at-risk stages suggests that early detection of individuals presenting with suggested risk factors for the development of BD is feasible in help-seeking young people. Future research should further develop/test stage-specific prevention and early targeted intervention approaches that were described in a naturalistic setting.

## Background

Bipolar disorders (BD) are severe mental disorders that are associated with an early onset and substantial psychosocial impairment (Carlson et al. 2000; Perlis et al. 2004). According to the World Health Organization Global Burden of Disease Study, BD rank within the top 10 causes of disability among all medical conditions worldwide, and about 3 million people suffer from BD in the European Union (Vos et al. 2012; Wittchen et al. 2011).

First signs of emerging BD frequently occur in early adolescence, and manifestation of the full disorder (at least one depressive and one (hypo-)manic/mixed episode) typically occurs in late adolescence or early adulthood (Carlson et al. 2000). Early onset BD is associated with greater rates of comorbid disorders (e.g., substance abuse), more recurrences, shorter periods of euthymia and a greater likelihood of suicide attempts (Perlis et al. 2004). Diagnosis of a first episode and adequate treatment are often delayed (up to ten years) (Baldessarini et al. 2003; Pfennig et al. 2011). Reasons for delayed diagnosis and treatment include: i) the natural development of the disease with depressive episodes as the first manifestation in about half of the patients (frequently leading to a diagnosis of unipolar depression), ii) nonspecific symptoms in early stages that are not classified as bipolar symptomatology, iii) suboptimal health care access and provision, and iv) fear of stigmatization by affected subjects (Leopold et al. 2012; Faedda et al. 2019). Treatment delay is associated with an impaired age-appropriate development, worse social adjustment and functional outcome, more hospitalizations, higher comorbidity rates, elevated risk of suicide, and reduced response to mood stabilizing drug treatment (Chen and Dilsaver 1996; Goldberg and Ernst 2002; Kessing et al. 2014; Matza et al. 2005; Miller et al. 2014; Post et al. 2010). Thus, early recognition of at-risk persons and early BD and primary interventions before the onset of the disorder and at early stages of BD are promising avenues for improving outcomes of the disorder (Collins et al. 2011; Forsman et al. 2014, 2015) in line with clinical staging models (Berk et al. 2017). Prevention of BD can target asymptomatic subgroups of individuals with risk factors for BD (selective prevention) or individuals with subthreshold symptoms that do not meet diagnostic BD criteria (indicated prevention, Fusar-Poli et al. 2019). For example, due to its high heritability (Craddock and Sklar 2009), a positive family history for BD is one of the major criteria for selective prevention of BD (Duffy et al. 2010, 2014; Mendlewicz and Rainer 1977; Smoller and Finn 2003). However, since many patients with BD do not have a positive family history, efforts are being made to develop clinical at-risk criteria so as to improve 

Prevention of BD (Axelson et al. 2015; Bechdolf et al. 2010; Brietzke et al. 2012; Correll and Olvet et al. 2014; Duffy et al. 2017; Faedda et al. 2014; Hafeman et al. 2016; Howes et al. 2010; Hunt et al. 2016; Leopold et al. 2012; Luby and Navsaria 2010; van Meter et al. 2016; Singh 2015; Skjelstad et al. 2010). In a systematic literature review, Rios and colleagues postulated a clinical staging model and determined stage I as a symptomatic period that can last up to years preceding the first manifest manic episode/BD. The first manifest manic episode/BD itself was defined as stage II (Rios et al. 2015). Early symptoms include (sub-)syndromal major depressive disorder/changes in mood/affective liability, fearfulness/anxiety, sleep disturbances, anger/irritability, and functional impairment (Bechdolf et al. 2010; Leopold et al. 2012; Correll and Hauser et al. 2014; van Meter et al. 2016; Faedda et al. 2019). Furthermore, longitudinal studies suggest a progression of psychopathology over time (Duffy et al. 2017; Hafeman et al. 2016), with symptoms usually becoming more specific and more similar to those of manifest BD (Axelson et al. 2015; van Meter et al. 2016; Pfennig et al. 2017; Tohen et al. 2003).

Based on this knowledge and clinical staging models, some semistructured instruments have been developed to identify young people at clinical risk for BD and to allow indicated prevention (i) the Early Phase Inventory for bipolar disorders (EPI*bipolar*, Leopold et al. 2012); (ii) the Bipolar Prodrome Symptom Scale (BPSS-FP, Correll and Olvet et al. 2014); (iii) Bipolar at-risk (BAR) criteria, Bechdolf et al. 2012/ the Semistructured Interview for Bipolar At Risk States, SIBARS, Fusar-Poli et al. 2018). Cross-sectional studies revealed promising results on the internal reliability and consistency of these instruments (Correll and Olvet et al. 2014; Bechdolf et al. 2012). Emerging longitudinal studies reported a transition risk to BD of 14% at 1-year (Bechdolf et al. 2014) and 23% at 2-years (Fusar-Poli et al. 2018), and adequate prognostic accuracy for conversion to BD (0.7 at 18 months, Fusar-Poli et al. 2018). Research is ongoing to validate the findings in larger cohorts.

Following early and appropriate detection, prevention of illness progression becomes crucial. Therefore, increasing research efforts are being targeted towards the development and evaluation of early selective and indicated interventions (McNamara et al. 2012). Choosing the appropriate intervention requires careful balancing risks and benefits in a shared decision process. Preliminary clinical category models and treatment guidance have been formulated based on emerging evidence and clinical expertise (Berk et al. 2007; Kapczinski et al. 2009; Leopold and Pfeiffer et al. 2013; Leopold and Pfennig et al. 2013; Chia et al. 2019). The basic proposed approach encompasses psychoeducation, counselling on illicit drug use, improvement of sleep quality and stable social rhythms, and further mental health strategies on preserving the young person’s ability to meet age-appropriate developmental tasks (Berk et al. 2007; Kapczinski et al. 2009; Leopold and Pfeiffer et al. 2013). Additionally, psychotherapy is an early intervention strategy, since (i) early symptoms (such as mood swings, disruptions of diurnal rhythm/ sleep disturbances) may be especially responsive to psychotherapy, (ii) it has an exceptionally favorable benefit/risk ratio, and (iii) is usually more acceptable to young patients compared to pharmacotherapy. Family focused psychotherapy tailored to the needs of the young at-risk subjects was associated with reduction of depressive symptoms, increase of psychosocial functioning and a longer time in remission (Miklowitz et al. 2013). Additionally, first data from small, open and uncontrolled studies show that Interpersonal and social rhythm therapy (IPSRT, Goldstein et al. 2014) led to better sleep, and Mindfulness-based cognitive therapy (MBCT, (Cotton et al. 2016; Strawn et al. 2016) to better emotion regulation and less anxiety. However, IPSRT plus informed referral were not significantly better compared to informed referral only in a succeeding small study (Goldstein et al. 2018). Recently published first data from early tailored cognitive behavioral group therapy applied to at-risk subjects show a reduction of affective symptoms and better psychosocial functioning after up to six month. However, there was no significant difference compared to the unstructured control group treatment (Leopold et al. 2020).

Evidence on pharmacological interventions in persons at-risk for BD is scarce (Chang et al. 2003; DelBello et al. 2007; Findling et al. 2007; Geller et al. 1998). Pharmacological treatment approaches with mood stabilizers (lithium and divalproex) for treatment of at-risk patients have only been investigated in small-sample studies not showing effectivity over placebo (Findling et al. 2007; Geller et al. 1998). In a larger sample aripiprazole (n = 30) was significantly better over 12 weeks in improving manic symptomatology and global clinical illness severity compared to placebo (n = 29, Findling et al. 2017).

Thus far, treatment guidelines do not contain specific recommendations on interventions of patients at-risk for BD (DGBS and DGPPN 2019), and no consistent recommendations for early stage disorder, except with respect to the indications for maintenance medication treatments (Chia et al. 2019). Additionally, clinical practice is poorly examined. Initiatives for the early detection of BD have been started to clarify whether early detection of at-risk stages for the development of BD is possible and which early intervention strategies may be most appropriate (Pfennig et al. 2012; Faedda et al. 2019).

Taken together, evidence for a progression of BD across early at-risk states is growing, and it is hypothesized that symptoms become more specific and similar to those of manifest BD over time (Axelson et al. 2015; Duffy et al. 2017; Hafeman et al. 2016; van Meter et al. 2016; Pfennig et al. 2017). However, clinical at-risk stages for BD are poorly examined regarding clinical characteristics/phenomenology, attendant symptoms and comorbid psychiatric disorders. Moreover, detail on the psychiatric and psychotherapeutic care offered to persons at risk for BD is largely missing. Clinical data from Early Detection and Intervention Center are useful to gain insight into clinical characteristics, care and treatment histories as well as treatments recommended and provided in early stages of developing BD. Based on clinical data of young people seen during the first nine years of the Early Detection and Intervention Center at the university hospital in Dresden, this analysis aimed to thoroughly describe different clinical at-risk constellations for BD with regard to individual risk factors and (comorbid) psychiatric disorders. Moreover, treatment history as well as treatment recommendations by the service were examined.

## Methods

### Design

This analysis was conducted in accordance to the REporting of studies Conducted using Observational Routinely-collected health Data (RECORD) statement (Benchimol et al. 2015). All data were collected as part of the service routine process. The analysis was in accordance to the APA ethical standards (American Psychiatric Association 2003) and the Declaration of Helsinki (World Medical Association 1964) and approved by the Ethics Committee of the Medical Faculty of the Technische Universität Dresden (EK290082014). All participants provided informed consent.

### Study population and setting

The analysis comprised sociodemographic, clinical and psychopathological characteristics as well as previous treatment experiences of help-seeking adolescents and young adults that consulted the Early Detection and Intervention Center at the university hospital Dresden, Germany, from May 2009 to April 2018. In addition, treatment recommendations given by the service specialists after multi-professional case conferences discussed with the clients in the shared decision process were analyzed.

### Proceeding at the Early Detection and Intervention Center Dresden

The Early Detection and Intervention Center Dresden was established in 2008 for help-seeking young people aged 15 to 35 years. It is open to clients with a broad symptomatology and/or need for counselling, and employs staff specifically trained in the early detection and treatment of BD (https://www.ddfruehdran.de/). Being a secondary care service, individuals can consult the service directly or via referral by psychologists, psychiatrists, primary care physicians or counselling services. Patients are assessed by clinical psychologists and/or psychiatrists in a comprehensive standardized, stepped diagnostic procedure, including early recognition instruments, if indicated. Diagnostic information is evaluated in multi-professional case conferences by experienced, specialized service staff members. Treatment decisions are based on the best available evidence. The content of the particular treatment recommendations does not follow a specific protocol but is given naturalistically based on the clinical experience of the service clinical experts, published evidence and clinical guideline recommendations as appropriate (Leopold et al. 2014). Recommendations might cover pharmacotherapy, psychotherapy, sociotherapy and other (e.g. additional specialized diagnostics) and can be administered in an outpatient, day care or inpatient setting, both at our university hospital and other hospitals or resident clinicians and psychotherapists. Clients and staff engage in a shared-decision process to agree upon the option most appropriate for the client at that point in time. In case patients already fulfill the diagnostic criteria for manifest BD, specific treatment is offered at the in- and outpatient facilities of the department of psychiatry at the same institution.

### Study measures, follow-up and clinical outcomes

At the initial contact, sociodemographic characteristics and reasons for visiting the service are assessed. If a diagnostic procedure is offered after the initial case conference, a comprehensive structured history is taken, including information about family history of psychiatric disorders and past treatment (incl. history of in- or outpatient (child-)psychiatric treatment, psychotherapy, pharmacotherapy with antidepressants, mood stabilizer and/or antipsychotics). History of substance use includes frequency of use of nicotine, alcohol, cannabis, amphetamines, hallucinogens, and cocaine (never, once, sporadically, frequently).

After obtaining a detailed psychiatric history and assessment of psychopathology, the German version of Structured Clinical Interview for DSM-IV Axis I disorders (SCID-I, First and Spitzer et al. 1997) is conducted by a different staff member that blinded to the results of the initial diagnostic interview to prevent premature assumptions and to ensure the patient is evaluated by two staff members (four-eye-principle). The SCID-I is a semi-structured clinical interview administered by trained psychiatrists/ psychologists and designed to yield psychiatric diagnoses consistent with DSM-IV/DSM-IV-TR (American Psychiatric Association 2007) diagnostic criteria. The duration of administration ranges from 30 to 120 min. The SCID-I possesses very good psychometric properties (First and Spitzer et al. 1997). To reach a good reliability regular rater trainings should be provided (Gorgens 2018). The reach a good validity the interview should be applied according to the LEAD procedure including longitudinal assessment performed by expert diagnosticians using all data that are available about the subject (Spitzer 1983). As part of the quality assurance of our Early Detection and Intervention Center we regularly train our diagnosticians and conduct all diagnostics according to the LEAD procedure.

When the development or manifestation of a personality disorder is suggested in the diagnostic process, the German version of Structured Clinical Interview for DSM-IV Axis II personality disorders (First and Gibbon et al. 1997) is applied.

Subjects with any suggestion of an at-risk state for BD, such as mood swings, and/or positive family history for affective or schizoaffective disorders, are additionally assessed with the following two instruments:

The Early Phase Inventory for Bipolar Disorders *(*EPI*bipolar*, Leopold et al. 2012) is a semi-structured interview comprising the categories disturbances in sleep and circadian rhythm, mood swings and affective lability, fearfulness and anxiety, psychosocial functioning, course of illness, former or current behavioral problems, and/or attention-deficit/hyperactivity disorder (ADHD) and substance use. Specific sleep and circadian rhythm disturbances were pre-defined and included for instance a high variability in sleeping times without external causation, phases of feeling fresh despite substantially less sleep than usual and having substantial problems with time zone changes. Specific features of the first depressive episode in disease history were pre-defined and included for instance substantial suicidal behavior, hypersomnia and hyperphagia. Additional data from history taking, including family history and data from the Bipolar Prodrome Symptom Scale-Full Prospective (BPSS-FP) completes the information. The BPSS-FP (Correll and Olvet et al. 2014) is a semi-structured interview with sections rating subthreshold (hypo-)manic, depressive and general symptoms. Patients fulfilling at least moderate severity in two or more (hypo-)mania items (attention and increased psychomotor activity are counted as one symptom) meet operationally defined criteria for a (hypo-)mania risk state. The BPSS-FP Mania scale has good to excellent psychometric properties in terms of internal consistency (Cronbachs α: 0.87) and interrater-reliability (intraclass correlation coefficient, ICC: 0.934) and satisfying scores for convergent validity (e.g. with the Young Mania Rating Scale) (Correll and Olvet et al. 2014).

Pre-defined *main* risk factors within EPI*bipolar* assessed within the above mentioned semi-structured interviews are I) a family history of BD, II) increasing cyclothymic mood swings with increased activity, and III) a (hypo-)mania risk state (at least moderate severity score in at least two (hypo-)mania items, taken from the BPSS-FP). *Secondary* risk factors are assigned to clusters A and B, that is: A1) specific sleep and circadian rhythm disturbances, A2) increasing cyclothymic mood swings without increased activity, A3) depressive episodes with specific features, B1) family history of schizophrenia, schizoaffective disorder or major depression, B2) lifetime diagnosis of depressive disorder, B3) lifetime diagnosis of ADHD, B4) decreased psychosocial functioning (work/school, social life, family or global), B5) episodic course of illness psychopathology, B6) increasing and periodic substance use of alcohol and/or cannabis. The EPI*bipolar* at-risk status was defined as presenting with (a) at least one secondary risk factor of each cluster (A & B), (b) at least one main risk factor and at least one secondary risk factor, or (c) at least two main risk factors. The EPI*bipolar* at-risk status was used in the analysis in order to generate baseline data for validation of the interview developed at our research group. It has been designed in cooperation with Dr. Correll to complement the BPSS-FP and capture very early signs of developing BD.

### Statistical analysis

All data were sampled in the project database of the Early Detection and Intervention Center Dresden (employing ACCESS). Routinely, data quality and completeness were checked; before analysis, data were cleaned. The software-package IBM SPSS Statistics Version 25 was used to compute descriptive statistics. Chi^2^-tests and t-tests were applied to test whether clinical and sociodemographic characteristics of subjects not at-risk/no manifest mania/BD (N = 433) vs. at-risk for or manifest mania/BD (N = 149) differed.

For the subsequent analyses, the following groups of patients were defined according to the considered pre-defined risk factors for BD:

- Group 1 (AR): EPI*bipolar* at-risk status, no lifetime depression (SCID-I), mania risk state (BPSS-FP)
- Group 2 (AR + LD): EPI*bipolar* at-risk status plus lifetime depression (SCID-I), mania risk state (BPSS-FP)
- Group 3 [AR + (H)MR]: EPI*bipolar* at risk status plus (hypo-)mania risk state (BPSS-FP), with or without lifetime depression (SCID-I)
- Group 4 (M/BD): manifest mania/BD (SCID-I).

In addition to the calculation of pairwise group comparison (with Bonferroni correction) we also analyzed planned contrasts representing assumed increases in risk for BD using Chi^2^-tests and t-tests.

Two-sided statistical significance was evaluated at the 5% level for all analyses. We additionally report results that failed to reach significance as a trend (10% level).

## Results

### Patient sample

Altogether, 890 help-seeking adolescents and young adults were seen at the Early Detection and Intervention Center Dresden between May 2009 and April 2018. About a quarter of all contacts (N = 220, 25%) did not enter the full diagnostic process after the initial contact. Reasons were that clients (1) did not describe symptoms that would meet the diagnostic criteria for a psychiatric disorder or at-risk states (e.g., symptoms could be explained as heartsickness or grief, N = 29, 3%), (2) were already in adequate treatment (N = 11, 1%), (3) had to be referred to a more specific service right away (e.g. trauma care service, addiction counselling) (N = 175, 20%), or (4) presented with the necessity of immediate crisis intervention/ in-patient treatment (N = 5, 1%). Another 88 persons (10%) left the diagnostic process prematurely (e.g. did not show up to further appointments despite several offers).

**Fig. 1:**
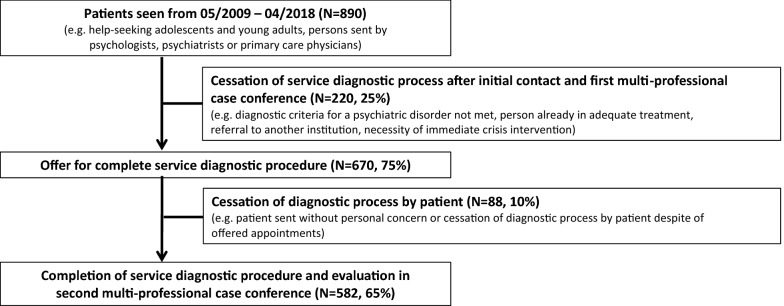
Subject flow chart

Overall, 582 (65%) patients completed the diagnostic procedure of the service and were included into the following analyses. The flow chart is presented in Fig. 1.

There were no significant differences regarding age and sex between subjects who did not enter the diagnostic process after the initial contact (N = 220; age: M = 25.0, SD = 10.2; female: N = 110, 50%), subjects who left the diagnostic process prematurely (N = 88; age: M = 25.2, SD = 8.3, female: N = 35, 40%), and subjects who completed the full diagnostic procedure (N = 582; age: M = 24.7, SD = 6.0, female: N = 279, 48%).

### Comparison of those with and without risk of BD

Table 1 presents sociodemographic and clinical characteristics of help-seeking adolescents and young adults who completed the diagnostic process (N = 582). Overall, 149 patients met any of the pre-defined risk factors for BD (N = 125, 21% of those with complete diagnostics) or received a diagnosis of manifest mania/ BD (N = 24, 4%).

**Table 1:** Sociodemographic and clinical characteristics of help-seeking adolescents and young adults completing the diagnostic process with vs without BD at-risk/manifest BD status (N = 582)

	Not at-risk for and no manifest mania/ Bipolar Disorder (N = 433)		At-risk for or manifest mania/ Bipolar Disorder (N = 149)		Group differences	P-value
	M	SD	M	SD	t	
Age	24.9	6.4	24.1	4.6	1.336	0.182
	**N**	**%**	**N**	**%**	**Chi**^**2**^	
Sex						
Female	194	44.9	85	57.0	**6.541**	**0.011**
Education						
Studies not finished (yet)	41	10.9	9	7.1	**13.663**	**0.008**
9th or 10th grade	142	37.8	37	29.4		
a-level	141	37.5	70	55.6		
University level	41	10.9	9	7.1		
No educational degree	11	2.9	1	0.8		
First contact to the health care system for present symptoms						
General practitioner or specialist physician	99	37.1	42	45.2	3.607	0.307
Counselling center	46	17.2	17	18.3		
Early Recognition Center	117	43.8	31	33.3		
Other	5	1.9	3	3.2		
Substance use						
Nicotine use^a^	138	35.6	64	45.4	**4.228**	**0.040**
Alcohol^a^	95	24.7	49	34.8	**5.271**	**0.022**
Cannabis use^a^	145	37.6	78	55.3	**13.33**	** < 0.001**
Amphetamine use^b^	48	12.4	23	16.3	1.332	0.249
Hallucinogen use^b^	24	6.2	18	12.8	**6.038**	**0.014**
Cocaine use^b^	21	5.4	12	8.5	1.697	0.193
Diagnoses according to DSM-IV (current or lifetime)						
Mental/behavioral dis. due to psychoactive substance use (F1x.x)	49	11.3	26	17.4	*3.715*	*0.054*
Mental/behavioral dis. due to use of alcohol (F10.x)	20	4.6	20	13.4	**13.424**	** < 0.001**
Mental/behavioral dis. due to use of cannabis (F12.x)	20	4.6	12	8.1	2.517	0.113
Mood (Affective) Disorders (3x.x)	175	40.4	108	72.5	**45.632**	** < 0.001**
Manic episode/Bipolar affective disorder (wo Hypomania) (F30.x-F31.x)	0	0.0	24	16.1	**72.745**	** < 0.001**
Depressive episode (F32.x)	80	18.5	27	18.1	0.009	0.923
Recurrent depressive disorder (F33.x)	79	18.2	51	34.2	**16.325**	** < 0.001**
Persistent mood (affective) disorders (F34.x)	25	5.8	6	4.0	0.671	0.413
Neurotic, stress-related and somatoform disorders (F4x.x)	136	31.4	42	28.2	0.542	0.462
Phobic anxiety disorders and other anxiety disorders (F40.x-F41.x)	99	22.9	31	20.8	0.271	0.603
Obsessive–compulsive disorder (F42.x)	16	3.7	6	4.0	0.034	0.855
Reaction to severe stress, adjustment disorder (F43.x)	23	5.3	8	5.4	0001	0.979
Eating disorders (F50x)	8	1.8	10	6.7	**8.750**	**0.003**
Specific, mixed & other personality disorders (F60.x-F61.x)	18	4.2	9	6.0	0.889	0.346
ADHD/ hyperkinetic disorder^c^(F90.x)	13	3.0	15	10.1	**12.082**	**0.001**
Comorbidity of above mentioned diagnoses						
0	147	33.9	22	14.8	**34.884**	** < 0.001**
1	194	44.8	62	41.6		
2 +	92	21.2	65	43.6		
History of child psychiatric treatment	35	8.1	24	16.1	**7.835**	**0.005**
Outpatient	20	4.6	11	7.4	1.679	0.195
Inpatient	19	4.4	18	12.1	**11.019**	**0.001**
History of psychiatric treatment/ psychotherapy	197	45.5	79	53.0	2.517	0.113
Outpatient	149	34.4	66	44.3	**4.649**	**0.031**
Inpatient	96	22.2	35	23.5	0.111	0.739
History of medication treatment						
Antidepressant	91	21.0	39	26.2	1.700	0.192
Mood stabilizer	4	0.9	4	2.7	2.535	0.111
Antipsychotic	60	13.9	12	8.1	*3.444*	*0.063*

*N* number, % percentage,* M* mean, *SD* standard deviation, *Chi*^2^Chi-square, t t-test, *p* p-value,

^a^never, once, sporadically vs. frequently; ^b^never, once vs.sporadically, frequently; ^c^including anamnestic information;

Patients at-risk for BD or with manifest mania/BD were of an equivalent age profile but more likely to be female (57% vs. 45%, p = 0.011). The educational profiles (representing the highest degree achieved) were somewhat different between the groups. Patients at-risk for BD or with manifest mania/BD reported significantly more frequent substance use (especially nicotine, alcohol, cannabis, and hallucinogens). They indicated significantly more often a preceding or comorbid mental disorder (especially recurrent depressive disorder, misuse of alcohol, eating disorder, and/or ADHD/ hyperkinetic disorder). Twice as many reported a history of (child-)psychiatric treatment (16% vs 8%, p = 0.005) and 44% vs. 34% (p = 0.031) had received outpatient treatment within adult psychiatry/ psychotherapy.

**Fig. 2:**
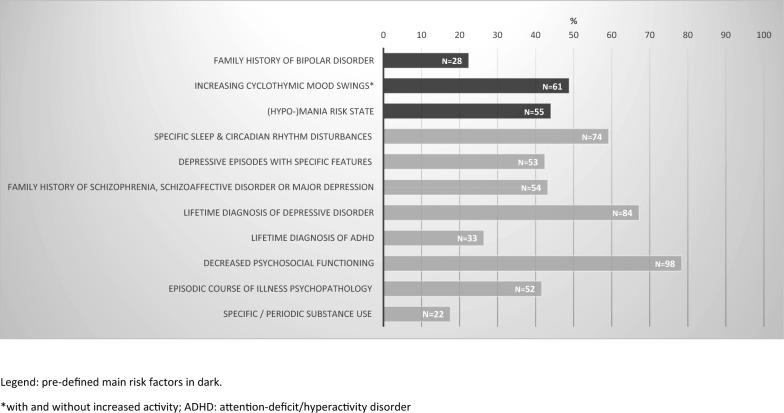
Distribution of a priori defined risk-factors for Bipolar Disorders according to EPI*bipolar* and BPSS (N = 125)

Figure 2 presents the rates and distribution of the pre-defined risk factors for BD according to EPI*bipolar* in at-risk patients (N = 125). Regarding the pre-defined *main* risk factors, 22% of at-risk patients presented with a family history of BD (first degree relative), 44% a (hypo-)mania risk state and 48% increasing cyclothymic mood swings with increased activity. From the *secondary* risk factors, decreased psychosocial functioning was the most frequent (78%), followed by lifetime diagnosis of depressive disorder (67%) and specific sleep and circadian rhythm disturbances (59%).

For the subsequent analyses, four groups of patients were defined as mentioned in the methods section according to the considered pre-defined risk factors for BD: AR (N = 21), AR + LD (N = 49), AR + (H)MR (N = 55), and M/BD (N = 24).

Table 2 presents sociodemographic and clinical characteristics of patients of the above-defined groups. Patients with manifest mania or BD (M/BD) were less often female compared to at-risk subjects (significantly compared to AR + LD, p = 0.036) and compared to the all subjects at-risk (p = 0.039). Substance use was common in all four groups with increasing frequency along the risk groups from AR to AR + (H)MR. Patients with mania/BD indicated more frequent cannabis and cocaine use compared to all at-risk subjects (cannabis by trend: p = 0.056, cocaine: p = 0.041). Manifest mental disorders were very common in all four groups, most frequently alcohol- or cannabis- related disorders, depression, anxiety and related disorders, and ADHD. The frequency of fulfilling at least two diagnoses apart from BD was increasing along the risk groups (AR 19%, AR + LD 49%, AR + (H)MR 47%).

**Table 2:** Sociodemographic and clinical characteristics for subjects in the four pre-defined groups (three BD at-risk and one manifest mania/BD) (N = 149)

	Group 1 AR (N = 21)		Group 2 AR + LD (N = 49)		Group 3 AR + (H)MR (N = 55)		Group 4 M/BD (N = 24)		Group differences with Bonferroni correction (significant results, only)^d^	p-value	Group differences for planned contrasts representing increasing risk for BD (significant results, only)^d^	p-value
	M	SD	M	SD	M	SD	M	SD				
Age	23.7	4.8	24.5	5.1	23.4	4.0	25.6	4.5				
	**N**	**%**	**N**	**%**	**N**	**%**	**N**	**%**				
Sex												
Female	12	57.1	33	67.3	32	58.2	8	33.3	G2 vs G4: Chi^2^ = 7.570	0.036	G1 + G2 + G3 vs G4: Chi^2^ = 6.565	0.039
Substance use												
Nicotine use^a^	9	42.9	21	44.7	22	41.5	12	60.0				
Alcohol^a^	4	19.0	16	34.0	21	39.6	8	40.0				
Cannabis use^a^	11	52.4	22	46.8	30	56.6	15	75.0			*G1* + *G2* + *G3 vs G4: Chi*^*2*^ = *3.652*	*0.056*
Amphetamine use^b^	4	19.0	5	10.6	9	17.0	5	25.0				
Hallucinogen use^b^	3	14.3	6	12.8	6	11.3	3	15.0				
Cocaine use^b^	1	4.8	2	4.3	5	9.4	4	20.0			G1 + G2 + G3 vs G4: Chi^2^ = 3.951	0.041
Diagnoses according to DSM-IV (current or lifetime)^#^												
Mental/behavioral dis. due to psychoactive substance use (F1x.x)	2	9.5	11	22.4	9	16.4	4	16.7				
Mental/behavioral dis. due to use of alcohol (F10.x)	1	4.8	9	18.4	7	12.7	3	12.5				
Mental/behavioral dis. due to use of cannabis (F12.x)	2	9.5	4	8.2	3	5.5	3	12.5				
Mood (Affective) Disorders (3x.x)	0	0.0	49	100.0	35	63.6	24	100.0	n.a		n.a	
Manic episode/Bipolar affective disorder (wo Hypomania) (F30.x-F31.x)	0	0.0	0	0.0	0	0.0	24	100.0	n.a		n.a	
Depressive Episode (F32.x)	0	0.0	20	40.8	7	12.7	0	0.0	n.a		n.a	
Recurrent depressive disorder (F33.x)	0	0.0	24	49.0	27	49.1	0	0.0	n.a		n.a	
Persistent mood (affective) disorders (F34.x)	0	0.0	5	10.2	1	1.8	0	0.0	n.a		n.a	
Neurotic, stress-related and somatoform disorders (F4x.x)	7	33.3	10	20.4	17	30.9	8	33.3				
Phobic anxiety disorders and other anxiety disorders (F40.x-F41.x)	4	19.0	6	12.2	15	27.3	6	25.0			*G1* + *G2vsG3* + *G4: Chi*^*2*^ = *3.406*	*0.065*
Eating disorders (F50x)	1	4.8	3	6.1	5	9.1	1	4.2				
Specific, mixed and other personality disorders (F60.x-F61.x)	3	14.3	3	6.1	3	5.5	0	0.0			*G1vsG2* + *G3* + *G4: Chi*^*2*^ = *2.928*	*0.087*
ADHD/ hyperkinetic disorder (F90.x)^c^	1	4.8	4	8.2	7	12.7	3	12.5				
Comorbidity of above mentioned diagnoses 0	11	52.4	0	0.0	11	20.0	0	0.0	G1 vs G2: Chi^2^ = 30.632 *G1 vs G3: Chi*^*2*^ = *8.655* G1 vs G4: Chi^2^ = 16.720 G2 vs G3: Chi^2^ = 11.913	p < 0.001 *p* = *0.078* p < 0.001 p = 0.018	G1vsG2 + G3 + G4: Chi^2^ = 27.810 *G1* + *G2* + *G3vsG4: Chi*^*2*^ = *5.340*	p < 0.001 *p* = *0.069*
1	6	28.6	25	51.0	18	32.7	13	54.2				
2 +	4	19.0	24	49.0	26	47.3	11	45.8				

*N* number, % percentage, *M* mean, *SD*: standard deviation, *Chi*^*2*^*:* Chi-square, p: p-value, *n.a*. not applicable, *AR* at-risk for bipolar disorder, *LD*: lifetime depression, *(h)MR* (Hypo-)Mania Risk State, *M/BD* Manifest Mania/ Bipolar Disorder, *G1* group 1*, G2 *group 2, *G3* group 3, *G4* group 4.

^a^never, once, sporadically vs. frequently; ^b^never, once vs. sporadically; ^c^ including anamnestic information; ^d^For reason of clarity we decided to present significant results only.

Statistics for non-significant results are available on request to inform future meta-analyses,

Table 3 presents the treatment history patients reported and treatment recommendations given by the service after the diagnostic process. There was no significant difference with regard to history of (child-)psychiatric treatment/ psychotherapy and history of drug treatment in the four groups. In all groups pretreatment with antidepressants was relatively common (14%-46%), whereas pretreatment with mood stabilizers was rare (< 5%). Planned contrasts revealed that patients with M/BD indicated more often a history of inpatient treatment (by trend: p = 0.077) as well as antidepressant (p = 0.017) and antipsychotic (p = 0.012) medication compared all at-risk persons.

**Table 3:** Treatment history and treatment recommendation of the service for subjects in the four pre-defined groups (three BD at-risk and one manifest mania/BD) (N = 149)

	Group 1 AR (N = 21)		Group 2 AR + LD (N = 49)		Group 3 AR + (H)MR (N = 55)		Group 4 M/BD (N = 24)		Group differences with Bonferroni correction (significant results, only)^a^	p-value	Group differences for planned contrasts representing increasing risk for BD (significant results, only)^a^	p-value
	N	%	N	%	N	%	N	%				
History of child psychiatric treatment	3	14.3	6	12.2	11	20.0	4	16.7				
Outpatient	2	9.5	2	4.1	4	7.3	3	12.5				
Inpatient	2	9.5	5	10.2	9	16.4	2	8.3				
History of psychiatric treatment/ psychotherapy	10	47.6	25	51.0	29	52.7	15	62.5				
Outpatient	8	38.1	22	44.9	24	43.6	12	50.0				
Inpatient	4	19.0	9	18.4	13	23.6	9	37.5			*G1* + *G2* + *G3 vs G4: Chi*^*2*^ = *3.124*	*0.077*
History of medication treatment												
Antidepressant	3	14.3	13	26.5	12	21.8	11	45.8			G1 + G2 + G3 vs G4: Chi^2^ = 5.722	0.017
Mood stabilizer	1	4.8	1	2.0	1	1.8	1	4.2				
Antipsychotic	2	9.5	2	4.1	3	5.5	5	20.8			G1 + G2 + G3 vs G4: Chi^2^ = 6.310	0.012
Treatment recommendation of service												
Psychotherapy (outpatient)	9	42.9	36	73.5	24	43.6	9	37.5	*G1 vs G2: Chi*^*2*^ = *6.000* G2 vs G3: Chi^2^ = 9.499 G2 vs G4: Chi^2^ = 8.815	*0.084* 0.012 0.018	G2 + G3 vs G3 + G4: Chi^2^ = 7.541	0.024
Psychiatrist (outpatient)	2	9.5	21	42.9	10	18.2	9	37.5	G1 vs G2: Chi^2^ = 7.404 G2 vs G3: Chi^2^ = 7.541	0.042 0.036		
Psychiatric institutional outpatients' department (PIA)	1	4.8	8	16.3	10	18.2	12	50.0	G1 vs G4: Chi^2^ = 11.157 G2 vs G4: Chi^2^ = 9.183 G3 vs G4: Chi^2^ = 8.419	0.006 0.012 0.024	*G1 vs G2* + *G3* + *G4: Chi*^*2*^ = *3.819* G1 + G2 vs G3 + G4: Chi^2^ = 5.062 G1 + G2 + G3 vs G4: Chi^2^ = 14.799	*0.051* 0.024 < 0.001
Inpatient treatment	2	9.5	9	18.4	10	18.2	4	16.7				
Recommended medication of service												
Antidepressant	0	0.0	17	34.7	4	7.3	1	4.2	G1 vs G2: Chi^2^ = 9.623 G2 vs G3: Chi^2^ = 12.091 G2 vs G4: Chi^2^ = 8.081	0.012 0.006 0.024	G1 vs.G2 + G3 + G4: Chi^2^ = 4.235 G1 + G2vsG3 + G4: Chi^2^ = 9.509	0.040 0.002
Mood stabilizer	0	0.0	3	6.1	5	9.1	9	37.5	G1 vs G2: Chi^2^ = 9.844 G2 vs G4: Chi^2^ = 11.547 G3 vs G4: Chi^2^ = 9.249	0.012 0.006 0.012	*G1 vs G2* + *G3* + *G4: Chi2* = *3.148* G1 + G2 vs G3 + G4: Chi^2^ = 6.629 G1 + G2 + G3 vs G4: Chi^2^ = 19.267	*0.076* 0.010 < 0.001
Antipsychotic	2	9.5	0	0.0	3	5.5	1	4.2				

*N *number, % percentage, *M* mean, *SD *standard deviation, *Chi*^*2*^Chi-square, *t* t-test, *p* p-value, * n.a.* not applicable,* AR* at-risk for bipolar disorder,* LD* lifetime depression,* (h)MR* (Hypo-)Mania Risk State,* M/BD* Manifest Mania/ Bipolar Disorder, G1: group 1, G2: group 2, G3: group 3, G4: group 4.

^a^For reason of clarity we decided to present significant results only.

Statistics for non-significant results are available on request to inform future meta-analyses

Patients of the four groups received differential treatment recommendations by the service. In subjects of the AR + LD group significantly more frequently outpatient psychotherapy (74%), outpatient psychiatric treatment (43%) and medication with antidepressant drugs (35%) was recommended compared to the other groups. Every second person diagnosed with manifest M/BD (50%) was referred to the in-house outpatients' department with specialization on BD treatment. The remaining persons with manifest mania/BD were offered inpatient treatment (17%) or psychotherapeutic/psychiatric outpatient treatment (38%). Mood stabilizers were recommended with increasing frequency from the AR to the M/BD group (0%, 6%, 9% and 38%).

## Discussion

This analysis examined sociodemographic, clinical and psychopathological characteristics as well as treatment histories of help-seeking persons who were seen at the Early Detection and Intervention Center Dresden from 2009 to 2018. Based on the characteristics, differential at-risk groups for BD were compared and treatment recommendations analyzed. The at-risk sample of our cohort is distinct from offspring samples of BD patients who were focused on in risk research so far (Canadian offspring study, Duffy et al. 2010; Pittsburgh Bipolar Offspring Study, BIOS, Birmaher et al. 2010; Dutch Bipolar Offspring Cohort Study, Wals et al. 2001 and Preisig et al. 2016).

The main findings of the analysis are:

- About one in five young help-seeking clients that completed the diagnostic process met the at-risk definition for bipolar disorders.
- Of the pre-defined main risk factors, increasing cyclothymic mood swings with increased activity was present in about half of the at-risk patients, a (hypo-)mania risk state in about forty percent, a positive family history in only about one in five. Decreased psychosocial functioning (about four in five), lifetime depression (two thirds) and specific sleep and rhythm disturbances (about 60%) were the most frequent secondary risk factors.
- Manifest diagnoses apart from BD were present in the majority of at-risk patients with increasing frequency of at least two diagnoses along the risk groups. Substance use was very common in at-risk patients and highest in patients diagnosed with manifest mania/BD, with use of cannabis in up to three quarters and alcohol in up to 40%.
- Treatment history was rather similar across groups.
- Regarding treatment recommendations, psychotherapy was frequently recommended in subjects at-risk for BD with a lifetime depression. Specialized treatment, including mood stabilizers, was recommended most frequently in manifest mania/BD patients.

Given a lifetime prevalence of BD of about 3%, a substantial amount of persons (one in five) met the at-risk definition for BD in this help-seeking sample of adolescents and young adults. Data to relate this frequency to are rare. For instance, in the Orygen Youth Health study 11% (N = 59/559) of the clients fulfilled BAR criteria and did not meet the exclusion criteria (including an (un)treated hypomania, a psychotic episode or current/past mood stabilizer treatment, Bechdolf et al. 2014). Reasons for the higher frequency compared to the Australian help-seeking sample might be that we included patients older than 25 years and did not exclude those with a history of mood stabilizer treatment. Moreover, the EPI*bipolar* risk assessment interview used in our study includes more potential risk symptoms for BD.

About 22% of at-risk patients indicated a history of BD in a first degree relative. Whether or not at-risk patients will develop BD is being evaluated as part of our ongoing follow-up analyses. Studies in offspring cohorts reported that 9–22% of children of BD patients developed BD-spectrum disorders (Faedda et al. 2019). In our sample about four out of five help-seeking youth at potential risk did not report a positive family history. This renders the assessment and integration of psychopathological and other clinical information into the early detection process vital, as merely the monitoring of offspring will not detect all people at-risk for BD. Early detection and intervention services that are able to offer low-threshold and comprehensive diagnostic procedures to help-seeking young people, as “Dresden early on”, could undertake this challenge.

Data is scarce regarding the prevalence of individual at-risk symptoms for the development of BD in help-seeking patients. A recent meta-analysis including 11 studies of differential cohorts revealed that the most common symptoms before the first mood episode were too much energy (68%), diminished ability to think, (63%), indecisiveness (62%), pressured speech (60%), talkativeness (60%), elated mood (58%), academic or work difficulties (56%), insomnia (54%, coded separately from decreased need for sleep), depressed mood (53%), and over-productive/goal-directed behavior (50%) (van Meter et al. 2016). The findings are in part overlapping with our data with respect to e.g. subthreshold hypomanic symptoms and depression. Additionally, sleep disturbances were seen in equal frequency and academic or work difficulties in more than half of our at-risk patients, too. We also observed an overlap with psychosis risk syndromes, e.g. regarding depressive symptoms and those related to anxiety (Fusar-Poli et al. 2020).

The finding of substance use in a high proportion of at-risk patients and in patients diagnosed with manifest mania/BD (with cannabis and alcohol being the most frequent substances) is in line with published evidence (Brietzke et al. 2012; Goldstein et al. 2013; Leopold et al. 2012; van Meter et al. 2016). Results are indicative of an early onset of substance misuse or substance use disorders in persons who later manifest BD (Beesdo-Baum et al. 2015). Patients with substance use disorders usually show low utilization of mental health care services (Mack et al. 2014). The Early Detection and Intervention Center Dresden with its low-threshold service seems to be an early opportunity to reach individuals at-risk for BD who present with unfavorable substance use habits. Drug counselling was recommended by our service in all identified cases and was part of the recommended psychotherapy. Overall, the frequency of substance use in subjects was higher compared to those published in 2008 for a German help-seeking sample of the Early Recognition and Intervention Center Cologne (Germany) (4–5%) (Schultze-Lutter et al. 2008). This difference might be partially due to varying diagnostic procedures (with differences in the detail of screening) and clinical foci of the two institutions (e.g. the service in Cologne is specialized in the early recognition of psychosis whereas that in Dresden has a somewhat broader outreach) and due to a change in overall substance use rates in Germany during this period. In the above mentioned recent meta-analysis regarding psychosis risk syndromes, 27% of young people at risk for psychosis were cannabis users, a lower proportion compared to our risk-sample. It has to be kept in mind when comparing our rates of substance use to that in other samples that our clientele to a great extend did not present for drug use issues as there are local low-threshold community drug counselling services available that are highly responsive to that issues.

Treatment histories were rather similar in help-seeking persons with and without risk for BD. Interestingly, 50% of subjects who fulfilled any of the pre-defined at-risk factors for BD or even satisfied the diagnostic criteria for manifest BD reported a history of psychiatric treatment, including psychotherapy. However, the increased risk for BD was rarely recognized by the previous care provider and only few patients were referred to the Early Detection and Intervention Center Dresden to evaluate the suspected diagnosis of BD or subthreshold symptomatology. This finding can be partially explained by the fact that emerging BD often is characterized by heterogeneous and non-specific symptomatology (Leopold and Pfennig et al. 2013; Lambert et al. 2016; Hauser and Correll 2013). However, early recognition of BD is very important as persons at-risk for BD or manifest BD/mania often suffer from symptoms such as sleep/circadian rhythm disturbances and functional impairment (60% and 80% of those persons in our sample). Additionally, antidepressant monotherapy was rather frequent in our at-risk subjects. This treatment approach should have been accompanied by close clinical monitoring because of its potential to generate or accelerate (hypo)manic symptomatology (DGBS and DGPPN 2019).

Psychiatric comorbidity in BD is often associated with a more severe course, poorer treatment adherence, and worse outcomes related to suicide and other complications. Additionally, it requires consideration when prescribing drugs (DGBS and DGPPN 2019). In line with previous evidence (Krishnan 2005), patients at-risk for BD or with manifest BD had significantly elevated rates of mental or behavioral disorders due to the (mis-)use of alcohol, recurrent depressive disorder, eating disorders and/or ADHD/hyperkinetic disorder. However, it is often unclear whether a co-occurring disorder is truly comorbid, a precursor of the disorder, a consequence of treatment, or a combination of those options (Faedda et al. 2019). Of the aforementioned comorbidities, that of eating disorders and BD seems the most underestimated in research and clinical service. There is recent evidence for higher rates of binge eating disorder and bulimia nervosa in BD (McElroy et al. 2016; Thiebaut et al. 2019). However, little is known about the timing of symptom onset, the individual trajectories of the disorders when co-occurring and the interaction of symptoms (Thiebaut et al. 2019). Data from a qualitative study suggest that in some patients symptoms of the eating disorder preceded the onset of BD (McAulay et al. 2021). Clinicians have to evaluate and monitor patients with BD and at-risk for BD for the presence and the development of any comorbid psychiatric condition to ensure appropriate interventions while avoiding potential iatrogenic complications. Moreover, clinicians should consider underlying or developing BD when evaluating individuals with other psychiatric diagnoses that often coexist with BD (not just unipolar depression). Anticonvulsants and other mood stabilizers may be especially helpful in treating BD patients with significant comorbidity (Krishnan 2005).

At the Early Detection and Intervention Center Dresden, no pre-defined treatment algorithm was followed and treatment recommendations were reached in a decision making process based on the clinical need, best available evidence and clinical expertise. Psychotherapy was recommended for the majority of subjects at-risk for BD, especially for patients who also met criteria for depression. Psychotherapy seems to be more acceptable to young patients at-risk compared to pharmacologic treatments (Rios et al. 2015). Preliminary studies of various psychotherapies, including psychoeducation strategies tailored specifically for BD in adolescents and young adults show promising results (Pavuluri et al. 2005; Pfennig et al. 2017) and should be considered when developing or revising evidence-based treatment guidelines for the early stages of BD.

Our results show that drug recommendation differed across the four at-risk BD groups. In at-risk youth with depression and no (hypo-)mania risk state (AR + LD), more frequently antidepressants were recommended. When a (hypo-)mania risk was present (AR + (H)MR), monotherapy with antidepressants was discouraged and mood stabilizers were recommended, the latter with the highest percentage in case of manifest mania/BD. These pharmacological recommendations are in line with current guideline recommendations. They reflect the favorable risk to benefit ratio of antidepressants compared to mood stabilizers in case of young people at risk for BD without (hypo-)mania risk state and the higher risk of instability and switch of antidepressant monotherapy when (hypo-)manic symptomatology becomes more present (DGBS and DGPPN 2019; Leopold and Pfennig et al. 2013).

Limitations: Although this analysis summarizes the experience of nine years work at the Early Detection and Intervention Center Dresden and provides clues for a better description and understanding of various risk constellations for BD, the following limitations have to be noticed: (1) data were collected in a clinical routine setting over a period of nine years. Within this period, progression regarding treatment recommendations and increasing clinical experiences of the service staff have to be considered. (2) Early detection instruments were not fully validated yet and psychometric properties need further examination (Correll and Olvet et al. 2014; Leopold et al. 2012). (3) Recall bias and inaccuracies regarding e.g. former treatment cannot be ruled out. Information on preceding mental disorders and treatment history can be biased or incomplete, since it was assessed retrospectively. However, illness history information is very important since preceding psychopathology is associated with the course of disease, and pretreatment experiences might be relevant for further health care utilization and treatment adherence (Duffy et al. 2017; Hafeman et al. 2016). (4) Another concern pertains to the phenotypic heterogeneity of at-risk states of BD. Due to the episodic character, the fluctuation of symptom severity and variations of functional impairment, early stages of BD are more complex and more difficult to identify than that of psychotic disorders (Brietzke et al. 2012; Duffy et al. 2017; Kafali et al. 2019; Rios et al. 2015; Hauser and Correll 2013). Non-specific symptoms may lead to false positive cases, and the typical overlap with potential preceding disorders and comorbidities further hampers adequate staging (Axelson et al. 2015; Duffy et al. 2017; Geller et al. 2002). However, help-seeking behavior and impairing symptomatology confirm the need of early diagnosis and individual treatment. (5) Thus far, this analysis only includes baseline data, a follow-up survey is underway to investigate long-term outcomes. (6) Finally, our findings stem from a sample of help-seeking adolescents and young adults. Results might not be generalizable to adolescents and young adults in general or to persons who decide not to engage in counselling, diagnostics or treatment.

## Conclusion

Despite the limitations inherent to the naturalistic research design and its descriptive character, in view of implications of the results for the long-term trajectory of the illness course this study clearly indicates the necessity of more research on stage-specific interventions, especially for the early phases of BD. A better understanding of the risk factors for BD and a clearer picture of the phenomenology of emerging BD offer hope for early identification and prevention (Pavuluri et al. 2005). Early detection and intervention of BD require detailed prospective examination in further studies, focusing on phenomenology of different at-risk states and examination of adequate, stage-specific treatment (Rios et al. 2015). Especially during early stages of BD, it is hoped that these research efforts will help provide data that guide clinicians towards tailored interventions with greater effectiveness in preventing or, at least, postponing the conversion to manifest BD. Clinical follow-up data could promote further development of evidence-based treatment approaches in early stages of BD (Berk et al. 2007; Kapczinski et al. 2009; Leopold and Pfeiffer et al. 2013). Studies on the feasibility, efficacy and cost-effectiveness of clinical approved interventions could be a next step (McGorry et al. 2006).

## Data Availability

The data sets generated and/or analysed during this study are not publicly available because participants did not agree to share their clinical data as an open source. They are, however, available from the corresponding author on reasonable request.

## Abbreviations

ADHD: Attention-deficit/hyperactivity disorder
APA: American Psychiatric Association
AR: EPI*bipolar* at-risk status
BAR criteria: Bipolar at-risk criteria
BD: Bipolar Disorder
BPSS-FP: Bipolar Prodrome Symptom Scale-Prospective
DSM-IV: Diagnostic and Statistical Manual of mental Disorders, 4th Edition
DSM-IV-TR: Diagnostic and Statistical Manual of mental Disorders, 4th Edition, Text revision
EPI*bipolar*: Early Phase Inventory for Bipolar Disorders
(H)MR: (Hypo-)mania risk state (BPSS-FP)
ICC: Intraclass correlation coefficient
IBM SPSS: Software package Statistical Package for the Social Sciences (SPSS) produced by International Business Machines Corporation (IBM)
LD: Lifetime depression (SCID-I)
M/BD: Manifest mania/ BD (SCID-I)
SCID-I: Structured Clinical Interview for DSM-IV Axis I disorders
SIBARS: Semistructured Interview for Bipolar At Risk States
